# Effect of a low-energy and enzyme-supplemented diet on broiler chicken growth, carcass traits and meat quality

**DOI:** 10.5194/aab-62-297-2019

**Published:** 2019-05-29

**Authors:** Elsayed O. S. Hussein, Gamaleldin M. Suliman, Alaeldein M. Abudabos, Abdullah N. Alowaimer, Shamseldein H. Ahmed, Mohamed E. Abd El-Hack, Mahmoud Alagawany, Ayman A. Swelum, Antonella Tinelli, Vincenzo Tufarelli, Vito Laudadio

**Affiliations:** 1 Department of Animal Production, College of Food and Agricultural Sciences, King Saud University, P.O. Box 2460, Riyadh 11451, Saudi Arabia; 2 Department of Meat Production, Faculty of Animal Production, University of Khartoum, Khartoum 1334, Sudan; 3 Department of Basic Sciences, College of Veterinary Medicine, Sudan University of Science and Technology, Khartoum, Sudan; 4 Poultry Department, Faculty of Agriculture, Zagazig University, Zagazig 44511, Egypt; 5 Department of Theriogenology, Faculty of Veterinary Medicine, Zagazig University, Zagazig 44511, Egypt; 6 Department of Veterinary Medicine, Section of Veterinary Pathology and Comparative Oncology, University of Bari “Aldo Moro”, Valenzano 70010, Bari, Italy; 7 Department of DETO, Section of Veterinary Science and Animal Production, University of Bari “Aldo Moro”, Valenzano 70010, Bari, Italy

## Abstract

The objective of this study was to evaluate the impact of a low metabolizable energy (low-ME) diet supplemented with a multienzyme blend (KEMZYME^®^) on the growth performance, carcass traits and meat quality of chickens. A total of 108 broiler chicks (Ross 308) were randomly allocated to three experimental groups with six replicates per treatment and five birds per replicate; the groups were treated as follows: a control diet with no additive and standard metabolizable energy (ME; 3200 kcal kg${}^{-1}$); a low metabolizable energy (low-ME; 3000 kcal kg${}^{-1}$) diet; and a low-ME diet + 0.5 g kg${}^{-1}$ diet of enzyme (low-ME–Enz). Live body weight (LBW) at 43 and 47 d and body weight gain (BWG) during the periods from 38 to 43, 43 to 47 and 33 to 47 d decreased with the low-ME and low-ME–Enz diets in comparison with the control-diet (p<0.05). The values of the feed conversion ratio (FCR) were significantly increased with low-ME diets with or without enzyme at all growing stages. There were no significant differences among treatments in terms of carcass traits. With the exception of the jejunum weight, dietary treatments did not affect any digestive tract segments. Meat hardness decreased with the low-ME–Enz diet compared with the other diets (P=0.039). Meat yellowness of the breast muscle increased (P=0.001) with the low-ME–Enz diet in comparison with the other treatments at 24 h post-slaughter. In conclusion, the low-ME diet supplemented with KEMZYME^®^ did not influence most of performance parameters and carcass traits of chickens; however, adding enzymes to the low-ME diet is an effective strategy to improve the meat quality criteria and small intestine characteristics.

## 1 Introduction

Maize (*Zea mays*) and soybean (*Glycine max*) meal (SBM) are major feedstuffs that provide energy and protein in commercial poultry diets (Zanella et al., 1990; Maisonnier-Grenier et al., 2004; Laudadio and Tufarelli, 2010), as both ingredients are considered to be highly digestible. The level of metabolizable energy (ME) of diets based on maize–SBM depends on the digestibility of nonstarch polysaccharides (NSP), starch and protein. Starch is the main source of energy in maize; however, complete digestion of maize starch in the digestive tract does not occur as some components are resistant to digestion (Brown, 1996; Tufarelli et al., 2007). Nevertheless, nondigestible SBM carbohydrates can be available to broiler chickens in presence of certain enzymes (Cowan, 1993). Therefore, enzyme-based strategies have been used to enhance the nutritional benefit of maize and SBM (Zanella et al., 1999; Maisonnier-Grenier et al., 2004).

Dietary supplementation with commercial enzymes as feed additives in poultry, to enhance the productive performance, is a well established feeding strategy (Alagawany and Attia, 2015; Abd El-Hack et al., 2017, 2018; Alagawany et al., 2017, 2018b). Zanella et al. (1999) found that adding a commercial enzyme to broiler chicken diets based on maize and SBM improved nutrient availability, digestibility and broiler performance. In addition, supplementation with enzyme enabled a reduced-energy diet to be adopted. On the contrary, other studies have reported that supplementing enzymes in the maize–SBM diet does not affect broiler chicken performance (Marsman et al., 1997; Kocher et al., 2002; Meng and Slominski, 2005; Alagawany et al., 2018a). KEMZYME^®^ is a multiple-enzyme product containing multiproteases, multiamylases and nonstarch polysaccharide (NSP) hydrolyzing enzymes, which has been specifically developed to improve nutrient availability and release extra amino acids and energy in multisubstrate broiler rations such as maize–SBM and wheat–SBM. Naqvi and Nadeem (2004) researched energy bioavailability of broiler diets using three levels of ME (3200, 3000 or 2800 kcal kg${}^{-1}$) after supplementation with KEMZYME^®^. However, little is known about the effect of commercial enzyme supplementation on meat quality and digestive organs, as well as on the sections of the digestive tract of broilers fed low-ME or normal-ME diets. Thus, the main objective of this study was to assess the effect of low- and normal-ME level maize–soybean-based diets supplemented with KEMZYME^®^ on the growth performance, meat quality, carcass traits and relative organ weights of broiler chickens.

**Table 1:** Ingredients and composition of diets fed to broiler chickens. (Min.–vit. premix refers to “mineral–vitamin premix”.)

Items	Control	Low-ME
Ingredients		
Maize grain	64.90	69.82
Soybean meal (48 % CP)	27.38	26.56
Palm oil	4.16	–
Dicalcium phosphate	1.45	1.44
Limestone	0.85	0.86
Salt	0.36	0.41
DL-methionine	0.24	0.23
L-lysine HCl	0.11	0.13
Choline Cl70	0.05	0.05
Min.–vit. premix (Arasco 0.5 %)	0.50	0.50
Nutrients${}^{*}$		
ME (kcal kg${}^{-1}$)	3200	3000
Dry matter (%)	89.62	89.17
Crude protein (%)	18.60	18.61
Arginine (%)	1.249	1.237
Lysine (%))	1.08	1.08
Methionine (%)	0.53	0.53
Cystine	0.31	0.32
Methionine + cystine (%)	0.85	0.85
Threonine (%)	0.708	0.706
Tryptophan (%)	0.226	0.224
Valine (%)	0.867	0.869
Ether extract (%)	6.89	2.92
Linoleic acid (%)	1.916	1.642
Crude fiber (%)	2.49	2.57
Calcium (%)	0.75	0.75
Total phosphorus (%)	0.59	0.594
Available phosphorus (%)	0.38	0.38
Sodium (%)	0.16	0.179

${}^{*}$Calculated according to NRC (1994).

## 2 Material and methods

### 2.1 Bird management and treatments

The experimental procedures were approved by the Local Animal Care and Ethics Committee of King Saud University, Riyadh, Saudi Arabia, ensuring compliance with EC Directive 86/609/EEC for animal trials.

A total of 108 broiler chicks (Ross 308) were randomly allocated to three treatments. Each group was divided into six replicates with six birds per replicate. The experiment was carried out in an environmentally controlled poultry unit within a temperature range of 22–24 ${}^{\circ }$C. Broiler chicks were raised in floor pens (1 m ×1 m) under similar management and hygienic conditions. Standard finisher diets (32–48 d) with an isonitrogenous content were offered in the form of maize–SBM mash (Table 1), and formulated to meet or exceed the nutrient requirements of birds (NRC, 1994). Additional enzyme supplementation was not included in the nutrient matrix (control diet). KEMZYME^®^ Plus is a multienzyme that combines three different NSP enzymes (cellulase, β-glucanase and xylanase) for the degradation of structural NSP, and two different endogenous-like enzymes (protease and amylase), to enhance the action of endogenous enzymes secreted in the gastrointestinal tract. KEMZYME^®^ Plus is a feed enzyme for piglets and poultry and improves the performance of animals when used as a supplement. Reformulation with KEMZYME^®^ Plus nutritional matrix minimizes feed costs by enabling less expensive feeds that have a higher fiber content and lower nutritional value to be used, when compared with more expensive feeds.

Upon arrival, the chicks received starter feed from 1 to 21 d, and then grower feed for the period from 22 to 31 d. Broilers were then distributed into the following treatments: a control diet with no additive and standard metabolizable energy (ME; 3200 kcal kg${}^{-1}$); a low metabolizable energy (low-ME; 3000 kcal kg${}^{-1}$) diet; and a low-ME diet + 0.5 g kg${}^{-1}$ diet of enzyme (low-ME–Enz).

### 2.2 Performance and carcass measurements

Feed intake was calculated on a daily basis by subtracting the amount of feed rejected from the feed offered. Body weight was measured every 5 d, and the feed conversion ratio (FCR) was computed for each group. At 48 d, a total of 12 birds from each treatment were randomly selected and processed to determine processing meat and carcass yields. Birds were put off feed for 10 h, then weighed and slaughtered, before being scalded and defeathered in a rotary picker. Heads and shanks were removed, and the remaining carcass was dissected to separate breast and leg. Similarly, fat, liver, intestines (duodenum, jejunum, ileum and ceca), heart, spleen, thigh and drumstick were separated and weighed. The percentage yield of each part was calculated on the basis of dressing weight (Poorghasemi et al., 2013).

**Table 2:** Effects of dietary treatments on the growth performance of broiler chickens.

	Treatments				
Items	Control	Low-ME	Low-ME–Enz	SEM	p value
Live body weight (BW; in grams)					
Day 33	1728	1735	1720	4.07	0.326
Day 38	2256	2232	2209	10.28	0.177
Day 43	2662${}^{a}$	2593${}^{b}$	2577${}^{b}$	13.42	0.012
Day 47	3116${}^{a}$	2993${}^{b}$	2980${}^{b}$	21.97	0.010
Body weight gain (BWG; in grams)					
Day 33–38	105.65	99.35	97.76	1.65	0.118
Day 38–43	101.38${}^{a}$	90.08${}^{b}$	91.91${}^{b}$	1.61	0.003
Day 43–47	90.88${}^{a}$	80.01${}^{b}$	80.73${}^{b}$	1.93	0.026
Overall mean	99.14${}^{a}$	89.76${}^{b}$	92.92${}^{b}$	1.53	0.008
Daily feed intake (in grams)					
Day 33–38	179	174	180	2.31	0.508
Day 38–43	169	170	166	1.24	0.400
Day 43–47	174	171	169	1.85	0.511
Overall mean	174	172	172	1.43	0.706
Feed conversion ratio (FCR; grams per gram)					
Day 33–38	1.70${}^{b}$	1.75${}^{a}$	1.85${}^{a}$	0.02	0.034
Day 38–43	1.67${}^{b}$	1.89${}^{a}$	1.81${}^{a}$	0.02	<0.001
Day 43–47	1.93${}^{b}$	2.14${}^{a}$	2.09${}^{a}$	0.03	0.010
Overall mean	1.89${}^{b}$	2.05${}^{a}$	2.04${}^{a}$	0.02	<0.001

Different superscripts within rows represent significant differences (p<0.05); SEM represents the standard error of the mean; overall treatment p value.

### 2.3 Meat quality

The breasts were anatomized, and both pectoralis muscles were weighed. The initial (at slaughter) and ultimate (after 24 h) hydrogen ion concentrations (pH${}_{i}$ and pH${}_{u}$, respectively) and the initial and ultimate color component values (color${}_{i}$ and color${}_{u}$, respectively) were determined. The pH was recorded using a pH meter (Model pH 211; Hanna Instruments, Woonsocket, RI, USA). Two readings were obtained for the breast muscle of each carcass, and the mean value of these measurements was the calculated. The color components of the CIELAB color system (1976) – $L^{*}$ (lightness), $a^{*}$ (redness) and $b^{*}$ (yellowness) – were measured using a Chroma meter (Konica Minolta CR-400; Konica Minolta, Tokyo, Japan) and were taken at two different locations on the top side of the breast muscle. An average of the two readings of the color components was used for statistical analyses. Following the measurements of pH and color quality, the breast muscles were stored frozen at -20 ${}^{\circ }$C for subsequent determination of cooking loss (CL) and shear force (SF). The water holding capacity (WHC) of the meat was measured according to the method described by Sun and Luo (1993). The frozen samples were then thawed overnight at 4 ${}^{\circ }$C, placed in a commercial countertop grill (Kalorik GR 28215; Kalorik, Miami Gardens, FL, USA), and cooked to an internal temperature of 70 ${}^{\circ }$C. The temperature was measured by introducing a thermocouple thermometer probe (Ecoscan Temp JKT; Eutech Instruments, Singapore) into the central core of the muscle. Muscles were weighed before and after cooking using a digital scale (Mettler MP1210; Mettler-Toledo Ltd., Leicester, UK) to determine the percentage CL as the difference between the initial and final weights. The cooked samples used for determining CL were reused to obtain SF according to Wheeler et al. (2005). Samples were cooled to room temperature (22 ${}^{\circ }$C), then cut into five 2.0 cm × 1.0 cm × 1.0 cm pieces, according to the methodology of Froning and Uijttenboogaart (1988). Shear force was determined as the maximum force (in kilograms) perpendicular to the fiber using a texture analyzer (TA.HD Stable Micro Systems; Stable Micro Systems Ltd., Godalming, UK) attached to a Warner–Bratzler knife. The crosshead speed was set at 120 mm min${}^{-1}$.

### 2.4 Statistical analysis

Data were subjected to an ANOVA using the general linear model (GLM) procedure in SPSS (SPSS, 1997). The differences between means were determined using the ANOVA procedure, followed by a Tukey post-hoc test to separate means. A p value of 0.05 was used to assess significance among means.

## 3 Results and discussion 

### 3.1 Growth performance

The effect of the dietary treatments on the broiler performance are reported in Table 2. Live body weight (LBW) at 43 and 47 d and body weight gain (BWG) during the periods from 38 to 43, 43 to 47 and 33 to 47 d decreased with low-ME and low-ME–Enz diets in comparison with the control (p<0.05). The values of the feed conversion ratio (FCR) were significantly increased with low-ME diets with or without enzyme, for all ages. There were no significant differences between the three treatments in terms of daily feed intake. The current study demonstrated that supplementation of enzyme in a low-ME diet had no major effect on any performance parameter. A reason for this may be that the study lasted only 16 d. Naqvi and Nadeem (2004) found that chickens fed the intermediate-level energy (3000 kcal kg${}^{-1}$) diet plus KEMZYME^®^ achieved better BWG and FCR values in comparison with those fed the same level of ME without KEMZYME^®^ supplementation; however, the BWG and FCR values were comparable to animals that were fed on the control diet (3200 ME kcal kg${}^{-1}$). Perić et al. (2008) investigated the effect of supplementation of an enzyme complex (containing amylase, protease, xylanase, β-glucanase, cellulose, pectinase and phytase) in broiler chicken diets on growth performance, and they found that enzyme supplementation had a positive effect on BWG and FCR. Zhou et al. (2009) found that the supplementation of broiler chicken diets with enzyme improved the utilization of ME, particularly in rations with lower ME levels. On the contrary, other researchers did not find any positive effects of dietary supplementation of enzyme for broiler chickens. Günal et al. (2004) found that the dietary supplementation of enzyme had no effect on BWG, dry matter intake, feed intake or the FCR of chickens. Similar results have been found in other studies. Live body weight, feed efficiency, feed intake and survivability of chickens were not significantly affected by dietary supplementation of enzyme (Sayyazadeh et al., 2006). Sherif (2009a) observed positive effects of some enzyme preparations (Natuzyme and Sicozyme) added to diets on the final LBW and BWG of broilers during the grower–finisher stage, but feed intake and FCR were unaffected. In another study, Sherif (2009b) reported that the addition of Avian Plus and Natuzyme enhanced the FCR and economic feasibility of broiler chickens fed plant protein sources, but feed intake and BWG were not influenced. Moreover, Cho et al. (2012) reported that feeding broilers with low-ME diets decreased the growth rate, and that these effects were alleviated by dietary supplementation of emulsifiers to the extent that growth was similar to that of birds fed high-ME diets.

**Table 3:** Effects of treatments on carcass yield and proportions of various carcass parts and organs (n=6).

	Treatments				
Items	Control	Low-ME	Low-ME–Enz	SEM	p value
Breast (g)	868.08	900.33	902.08	14.26	0.562
Carcass (%)	73.69${}^{b}$	75.03${}^{a}$	75.40${}^{a}$	0.26	0.018
Leg (g)	638.16	611.91	618.08	12.61	0.604
Drumstick (g)	24.01	23.33	22.75	3.03	0.614
Heart (g kg${}^{-1}$ SW)	16.25	17.75	18.16	0.46	0.213
Fat (g kg${}^{-1}$ SW)	35.58	37.16	34.75	1.41	0.789
Liver (g kg${}^{-1}$ SW)	69.33	65.83	64.75	0.46	0.387
Gizzard (g kg${}^{-1}$ SW)	73.50	67.91	63.33	1.88	0.084
Spleen (g kg${}^{-1}$ SW)	5.58${}^{b}$	7.01${}^{a}$	6.41${}^{a}$	0.15	<0.001
Duodenum (g)	33.41	33.83	32.83	0.63	0.614
Duodenum (cm)	33.41	33.83	32.83	0.63	0.822
Jejunum (g)	65.01${}^{a}$	53.08${}^{b}$	44.91${}^{c}$	2.34	0.001
Jejunum (cm)	86.33	82.41	82.41	1.52	0.491
Ileum (g)	57.08	55.41	49.33	1.50	0.083
Ileum (cm)	89.25	87.66	89.41	1.54	0.885
Ceca (g)	24.01	23.41	23.25	0.96	0.949
Ceca (cm)	21.66	21.33	21.50	0.37	0.939

Different superscripts within rows represent significant differences (p<0.05); SW represents slaughter weight.

### 3.2 Carcass traits and relative organ weights

Our findings indicated that there were no significant differences among the treatments in terms of carcass traits (Table 3). However, spleen weight and carcass yield were improved with the low-ME diets, either with or without enzyme, in comparison with the control. These results are in agreement with those of Holsheimer and Ruesink (1993), who found that carcass yields were not affected by gradual increases of ME (from 2750 to 3250 kcal kg${}^{-1}$ diet). Downs et al. (2006) reported similar results; they observed that dietary energy density did not influence carcass characteristics of broiler chicks. On the contrary, Mohammadigheisar et al. (2018) found that chickens fed a low-energy diet with multienzyme supplementation had the highest relative liver weights (p<0.05). Hidalgo et al. (2004) reported similar responses of carcass yield to increasing levels of ME in the rations of straight-run broilers. Sayyazadeh et al. (2006) concluded that abdominal fat and carcass yield of broiler chickens were not significantly influenced by supplementation of enzyme to wheat, maize and barley-based diets. Conversely, Bin Baraik (2010) found no effect of commercial enzymes, applied individually or in combinations, on carcass yield, dressing percent and weight of internal organs of broilers. They also observed that there were no statistical differences in the percentage of commercial cuts (drumstick, breast, wing and thigh). These results also agreed with the recent results obtained by Younis (2013).

**Table 4:** Effects of dietary treatment on the meat quality of broiler chickens.

	Treatments				
Item	Control	Low-ME	Low-ME–Enz	SEM${}^{1}$	p value${}^{2}$
Water holding capacity (%)	21.6	20.3	20.3	0.260	0.089
Myofibril fragmentation index	0.458	0.458	0.424	0.001	0.256
Cooking loss (%)	35.08	36.54	33.89	0.603	0.203
Shear force (kg)	1.65	1.63	1.24	0.118	0.287
Hardness (kg)	0.68${}^{a}$	0.68${}^{a}$	0.54${}^{b}$	0.020	0.039
Springiness index	0.70	0.69	0.72	0.001	0.310
Cohesiveness index	0.47	0.47	0.49	0.007	0.484
Chewiness index	0.23	0.22	0.19	0.009	0.209

Different superscripts within rows represent significant differences (p<0.05).

**Table 5:** Effects of dietary treatments on the color and pH of broiler chicken meat.

	Treatment				
Parameters	Control	Low-ME	Low-ME–Enz	SEM${}^{1}$	p value${}^{2}$
pH${}_{i}$ (initial value at slaughter)	6.51	6.45	6.65	0.033	0.108
pH${}_{u}$ (ultimate value after 24 h)	5.99${}^{b}$	5.98${}^{b}$	6.08${}^{a}$	0.01	0.032
Temperature (${}^{\circ }$C, at slaughter)	26.58${}^{a}$	25.51${}^{b}$	22.55${}^{c}$	0.35	<0.001
Color${}_{i}$ (initial value at slaughter)					
$L^{*}$	39.01	39.87	40.18	0.55	0.687
$a^{*}$	2.14	2.25	2.58	0.104	0.214
$b^{*}$	2.51	2.98	3.37	0.17	0.130
Color${}_{u}$ (ultimate value after 24 h)					
$L^{*}$	45.37	44.50	45.71	0.411	0.473
$a^{*}$	2.69	2.53	2.66	0.15	0.914
$b^{*}$	4.11${}^{b}$	4.30${}^{b}$	5.84${}^{a}$	0.22	0.001

Different superscripts within rows represent significant differences (p<0.05); $L^{*}$: lightness; $a^{*}$: redness; $b^{*}$: yellowness.

### 3.3 Intestinal segments

Dietary treatments did not affect digestive tract segments, apart from jejunum weight. Jejunum weight decreased under a low-ME or low-ME–Enz diet, when compared with the control (Table 3, p<0.001). To adapt to those changes, secretion activities of the intestine may increase, which, in turn, may lead to increases in the weight and size of the gastrointestinal tract, liver and pancreas. Increased size of the gastrointestinal tract and intestine could be adaptive responses to an increased need for exogenous enzymes (Brenes et al., 1993). On the contrary, Wang et al. (2005) showed that the length and weight of the ileum and the length of the cecum decreased (linearly, p<0.01) at 21 d of age with increasing dietary enzyme supplementation. Additionally, the length and weight of the ileum and the length of cecum decreased (linearly, p<0.05) as the enzyme level increased at 42 d of age. Moreover, at the ages of 21 and 42 d, relative weights of liver and pancreas decreased (linearly, p<0.01) with increasing enzyme level (Wang et al., 2005). Brenes et al. (1993) stated that the addition of supplemental enzymes to barley-based diets reduced the lengths of the jejunum, duodenum and ileum, but enzyme treatment had no significant effect on organ size in a wheat-based diet. In general, the use of commercial enzymes in the control diet or in the low-ME diet altered the morphology of the different segments of the gastrointestinal tract when compared with the control diet. Enzyme addition to broiler diets resulted in positive impacts on the energy digestibility of broilers (Pourreza et al., 2007). Xylanase supplementation significantly improved nutrient utilization and more nutrients were available to the poultry (Hosseini and Afshar, 2017; Tufarelli et al., 2007). Ramesh and Chandrasekaran (2011) reported that supplementation of enzyme improved the apparent metabolizable energy, and protein and NSP digestibilities in birds, which helped with better utilization of feedstuffs.

### 3.4 Meat quality criteria

Apart from meat hardness, no parameters of meat quality were statistically different among the treatments (Table 4). Hardness was lower with the low-ME–Enz diet when compared with the control and low-ME diets (P=0.039). In agreement with our results, Habib (2016) reported that the physical properties of broiler breast meat (pH and water holding capacity) were not significantly affected by enzyme supplementation (P>0.05). These results were also in agreement with the data obtained by Bin Baraik (2010), who observed no significant effect of commercial enzymes (xylanase and phytase), applied individually or in combination, on meat composition and meat quality values.

The results of the present study showed that the yellowness of breast muscle was increased (P=0.001) with the low-ME–Enz diet in comparison with the other treatments 24 h after slaughter. Lightness and redness were not influenced by dietary treatment, which is inconsistent with the data of Cho and Kim (2013) and Mohammadigheisar et al. (2018), who showed that feeding broiler chickens on low-energy diets resulted in a higher lightness value. On the contrary, the supplementation of multienzymes to low-energy diets decreased the lightness value. The results presented in Table 5 show that, 24 h after slaughter, the pH of the breast meat was affected (p=0.032) by treatments, and the low-ME–Enz diet had a higher pH (6.08). At slaughter, the pH value was not influenced by dietary treatments. However, the results of the current study contradict the findings of Wang et al. (2009), who found that dietary treatments had no effect on the pH of breast meat. The temperature at slaughter was significantly decreased with treatments when compared with the control (p<0.001).

## 4 Conclusions

Our data showed that a low-ME diet supplemented with KEMZYME^®^ did not affect most performance parameters and carcass traits of broiler chickens. However, live body weight at 43 and 47 d and body weight gain during the periods from 38 to 43, 43 to 47 and 33–47 d were significantly decreased with the low-ME and low-ME–Enz diets in comparison with the control. The values of the feed conversion ratio were significantly increased with low-energy diets with or without enzyme for all ages. Thus, adding enzymes to low-energy diets is an effective feeding strategy to improve the meat quality criteria and small intestine characteristics.

## Data Availability

The data sets are available upon request from the corresponding author.

## References

[bib1.bib1] Abd El-Hack ME, Alagawany M, Laudadio V, Demauro R, Tufarelli V (2017). Dietary inclusion of raw faba bean instead of soybean meal and enzyme supplementation in laying hens: Effect on performance and egg quality. Saudi J Biol Sci.

[bib1.bib2] Abd El-Hack ME, Chaudhry MT, Mahrose KM, Noreldin A, Emam M, Alagawany M (2018). The efficacy of using exogenous enzymes cocktail on production, egg quality, egg nutrients and blood metabolites of laying hens fed distiller's dried grains with solubles. J Anim Physiol Anim Nutr.

[bib1.bib3] Alagawany M, Attia AI (2015). Effects of feeding sugar beet pulp and avizyme supplementation on performance, egg quality, nutrient digestion and nitrogen balance of laying Japanese quail. Avian Biol Res.

[bib1.bib4] Alagawany M, Attia AI, Ibrahim ZA, Mahmoud RA, El-Sayed SA (2017). The effectiveness of dietary sunflower meal and exogenous enzyme on growth, digestive enzymes, carcass traits and blood chemistry of broilers. Environ Sci Pollut Res.

[bib1.bib5] Alagawany M, Attia AI, Ibrahim ZA, El-Hack MA, Arif M, Emam M (2018). The influences of feeding broilers on graded inclusion of sunflower meal with or without avizyme on growth, protein and energy efficiency, carcass traits and nutrient digestibility. Turk J Vet Anim Sci.

[bib1.bib6] Alagawany M, Elnesr SS, Farag MR (2018). The role of exogenous enzymes in promoting growth and improving nutrient digestibility in poultry. Iran J Vet Res.

[bib1.bib7] Bin Baraik BSS (2010). Effect of adding xylanase and phytase enzymes to broiler diets on performance and carcass yield and quality [dissertation].

[bib1.bib8] Brenes A, Smith M, Guenter W, Marquardt RR (1993). Effect of enzyme supplementation on the performance and digestive tract size of broiler chickens fed wheat- and barley-based diets. Poult Sci.

[bib1.bib9] Brown I (1996). Complex carbohydrates and resistant starch. Nutr Rev.

[bib1.bib10] Cho JH, Kim IH (2013). Effects of beta-mannanase supplementation in combination with low and high energy dense diets for growing and finishing broilers. Livest Sci.

[bib1.bib11] Cho JH, Zhao PY, Kim IH (2012). Effects of emulsifier and multi-enzyme in different energy density diet on growth performance, blood profiles, and relative organ weight in broiler chickens. J Agric Sci.

[bib1.bib12] Cowan WD (1993). Understanding the manufacturing, distribution, application, and overall quality of enzymes in poultry feeds. J Appl Poult Res.

[bib1.bib13] Downs KM, Lien RJ, Hess JB, Bilgili SF, Dozier III WA (2006). The effects of photoperiod length, light intensity, and feed energy on growth responses and meat yield of broilers. J Appl Poult Res.

[bib1.bib14] Froning GW, Uijttenboogaart TG (1988). Effect of post-mortem electrical stimulation on color, texture, pH, and cooking losses of hot and cold deboned chicken broiler breast meat. Poult Sci.

[bib1.bib15] Günal M, Yasar S, Forbes JM (2004). Performance and some digesta parameters of broiler chickens given low or high viscosity wheat-based diets with or without enzyme supplementation. Turk J Vet Anim Sci.

[bib1.bib16] Habib BB (2016). Effect of feed supplemented with xylam enzyme on performance, carcass characteristics and meat quality of broiler chicks. J Appl Vet Sci.

[bib1.bib17] Hidalgo MA, Dozier III WA, Davis AJ, Gordon RW (2004). Live performance and meat yield responses of broilers to progressive concentrations of dietary energy maintained at a constant metabolizable energy-to-crude protein ratio. J Appl Poult Res.

[bib1.bib18] Holsheimer JP, Ruesink EW (1993). Effect on performance, carcass composition, yield, and financial return of dietary energy and lysine levels in starter and finisher diets fed to broilers. Poult Sci.

[bib1.bib19] Hosseini SM, Afshar M (2017). Effects of feed form and xylanase supplementation on performance and ileal nutrients digestibility of heat-stressed broilers fed wheat-soybean diet. J Appl Poult Res.

[bib1.bib20] Laudadio V, Tufarelli V (2010). Treated fava bean (Vicia faba var. minor) as substitute for soybean meal in diet of early phase laying hens: Egg-laying performance and egg quality. Poult Sci.

[bib1.bib21] Maisonnier-Grenier S, Rouffineau F, Geraert PA (2004). Enzyme versatility: Key for efficacy in various feedstuffs and species.

[bib1.bib22] Mohammadigheisar M, Kim HS, Kim IH (2018). Effect of inclusion of lysolecithin or multi-enzyme in low energy diet of broiler chickens. J Appl Poult Res.

[bib1.bib23] Naqvi LU, Nadeem A (2004). Bioavailability of metabolizable energy through kemzyme supplementation in broiler rations. Pak Vet J.

[bib1.bib24] National Research Council (1994). Nutrient Requirements of Poultry.

[bib1.bib25] Perić L, Milošević N, Dukić-Stojčić M, Bjedov S, Rodić V (2008). Effect of enzymes on performances of broiler chickens. Biotechnol Anim Husband.

[bib1.bib26] Poorghasemi M, Seidavi A, Qotbi AAA, Laudadio V, Tufarelli V (2013). Influence of dietary fat source on growth performance responses and carcass traits of broiler chicks. Asian-Australas J Anim Sci.

[bib1.bib27] Pourreza J, Samie AH, Rowghani E (2007). Effect of supplemental enzyme on nutrient digestibility and performance of broiler chicks fed on diets containing triticale. Int J Poult Sci.

[bib1.bib28] Ramesh J, Chandrasekaran DC (2011). Effect of exogenous enzyme supplementation on performance of cockerels, Tamil Nadu. J Vet Anim Sci.

[bib1.bib29] Sayyazadeh H, Rahimi G, Rezaei M (2006). Influence of enzyme supplementation of maize, wheat and barley-based diets on the performance of broiler chickens. Pak J Biol Sci.

[bib1.bib30] Sherif KhEl (2009). Performance of broiler chicks fed plant protein diets supplemented with commercial enzymes. J Agric Sci Mansoura Univ.

[bib1.bib31] Sherif KhEl (2009). Effect of using probiotics and enzymes with plant-protein diets in broiler performance. J Agric Sci Mansoura Univ.

[bib1.bib32] SPSS (1997). SPSS, Base 7.5 for Windows.

[bib1.bib33] Sun YM, Luo M (1993). Livestock and Poultry Meat Science.

[bib1.bib34] Tufarelli V, Dario M, Laudadio V (2007). Effect of xylanase supplementation and particle-size on performance of guinea fowl broilers fed wheat-based diets. Int J Poult Sci.

[bib1.bib35] Wang JP, Hong SM, Yan L, Yoo JS, Lee JH, Jang HD, Kim HJ, Kim IH (2009). Effects of single or carbohydrases cocktail in low-nutrient-density diets on growth performance, nutrient digestibility, blood characteristics, and carcass traits in growing–finishing pigs. Livest Sci.

[bib1.bib36] Wang ZR, Qiao SY, Lu WQ, Li DF (2005). Effects of enzyme supplementation on performance, nutrient digestibility, gastrointestinal morphology, and volatile fatty acid profiles in the hindgut of broilers fed wheat-based diets. Poult Sci.

[bib1.bib37] Wheeler TL, Cundiff LV, Shackelford SD, Koohmaraie M (2005). Characterization of biological types of cattle (Cycle VII): Carcass, yield, and longissimus palatability traits. J Anim Sci.

[bib1.bib38] Younis MAE (2013). The utilization of (mesquite) Prosopis juliflora pods with xylanase and phytase enzymes in the broilers diets [dissertation].

[bib1.bib39] Zanella I, Sakomura NK, Silversides FG, Fiqueirdo A, Pack M (1990). Effect of enzyme supplementation of broiler diets based on maize and soybeans. Poult Sci.

[bib1.bib40] Zhou Y, Jiang Z, Lv D, Wang T (2009). Improved energy-utilizing efficiency by enzyme preparation supplement in broiler diets with different metabolizable energy levels. Poult Sci.

